# The Association of IL-12b Polymorphisms with Systemic Lupus Erythematosus in Chinese Han Population

**DOI:** 10.1155/2012/724872

**Published:** 2012-05-16

**Authors:** Yong Shao, Jie Zhang, Yuewen Chen, Qi Wu, Ming Guan, Bo Yu, Jun Wan, Wei Zhang

**Affiliations:** ^1^Shenzhen Key Lab for Translational Medicine of Dermatology, Shenzhen PKU-HKUST Medical Center, No. 1120, Lianhua Road, Futian District, Shenzhen, Guangdong 518036, China; ^2^Department of Dermatology, Shenzhen Hospital Peking University, Shenzhen, Guangdong 518036, China; ^3^Biomedical Research Institute, Shenzhen PKU-HKUST Medical Center, No. 1120, Lian-Hua Road, Fu-Tian District, Shenzhen, Guangdong 510632, China; ^4^Department of Clinical Laboratory, Huashan Hospital, Shanghai 200040, China; ^5^Division of Life Science, The Hong Kong University of Science and Technology, Hong Kong

## Abstract

*Background*. Systemic lupus erythematosus (SLE) is a complex immune disease. The genetic variation in the IL-12b gene was found to associate with SLE in Caucasian population. In this study, we examined this association in Chinese Han population by a recently developed method, unlabeled probe-based high resolution melting analysis. *Methods*. A total of 297 SLE patients and 351 controls were recruited. Unlabeled probe-based high resolution melting analysis (HRMA) was used in genotyping. *Results*. Statistically significant differences were observed in both genotype and allele frequencies for rs6887695 in the SLE patients as compared with the controls. Minor allele (C) of rs6887695 (*P* = 0.031, OR 0.78, [95% CI 0.63-0.98]) was found to be protective against SLE. The association of SNP rs6887695 with the diagnostic criteria of SLE was also examined. Minor allele (C) exerts protective effect on the incidence of arthritis (*P* = 0.013, OR = 0.65, 95% CI = 0.47-0.92) and abnormalities of antinuclear antibody (*P* = 0.022, OR = 0.68, 95% CI = 0.49–0.95). IL-12b SNPs were irrelevant to other diagnostic criteria of SLE. *Summary*. Polymorphisms of rs6887695 in IL-12b gene were associated with disease risk, as well as arthritis and antinuclear antibody synthesis, of systemic lupus erythematosus in Chinese population.

## 1. Introduction

Interleukin-12 (IL-12) is a heterodimeric cytokine that is produced during innate immune response by monocytes, macrophages, dendritic cells (DCs), neutrophils, and B cells [1, 2]. The general function of IL-12 is thought to be as an important immunomodulator linking innate recognition of pathogens to development of adaptive immune response [1, 3]. Interleukin (IL-12b), also known as p40, is one of the components of IL-12 which covalently combine with p35 (IL-12a) to form mature IL-12 protein. Otherwise, IL-12b could also interact with p19 (IL-23a) to form the other IL-12 family member, IL-23. IL-23 has strong capacity of induction of a novel subset of T cells, the T-helper-17 (Th17) cells. These novel T-helper cells are characterized by production of IL-17 cytokine which is strongly implicated in the pathophysiology of various autoimmune diseases [4]. Owing to the important role of IL-12 and IL-23 during the immune response, IL-12b, the common component of IL-12 and IL-23, might be a candidate gene of autoimmune disease. Indeed, IL-12b has been found to associate with several autoimmune diseases including psoriasis, rheumatoid arthritis, and type 1 diabetes [5–9]. 

Systemic lupus erythematosus (SLE) is a complex autoimmune disease with multiple organs affected. Autoantibody production, formation of immune complexes and tissue inflammation are the major clinical features of SLE. The reported prevalence of SLE is 20 to 150 cases per 100,000 with a female/male ratio of 9 : 1 [10, 11]. The etiology of SLE has not been completely understood, but it is well known that both genetic components and environmental factors contribute to the pathogenesis of SLE [12, 13]. Recently, a series of genome-wide association studies (GWAS) were employed for identifying SLE associated genetic variants [14–19]. These studies provided new insights into the understanding of this complex disease. In the Caucasian populations, it has been suggested that a polymorphism rs6887695 located on IL-12B gene was associated with SLE [19]. Here, we examined this association in Chinese Han population by a recently developed method, unlabeled probe-based high-resolution melting analysis. 

## 2. Methods

### 2.1. Patients

A total of 297 patients (27 males and 270 females; median age 29 years, range 12–55) who fulfilled the American College of Rheumatology criteria for SLE [20] and 351 ethnically matched healthy controls (31 males and 320 females; median age 28 years, range 17–46) were recruited from Shenzhen Hospital, Peking University. The control group had neither family history nor symptoms related to SLE. The study was approved by the institutional review board of the Shenzhen Hospital, and written informed consent was taken from all patients. 

### 2.2. Genotyping

Genomic DNA was isolated from peripheral blood cells by using Innogent genomic DNA extraction kit (Innogent, China) according to manufactory instruction. Genotyping was assayed by high-resolution melting with unlabeled probe as previously described [21]. Briefly, asymmetric PCR reaction was performed in a volume of 20 *μ*L containing 20 ng of genomic DNA, 1 × PCR buffer (Takara, Japan), 200 *μ*M dNTPs, 0.5 U rTaq DNA polymerase (Takara, Japan), 0.05 *μ*M forward primer, 0.5 *μ*M excess reverse primer, and 0.5 *μ*M C3-block probe. The PCR reactions were performed in an S1000 Thermal Cycler (Bio-Rad, USA); the conditions included an initial denaturation at 94°C for 2 min, followed by 45 cycles of 94°C for 30 s, 55°C for 30 s, and 72°C for 20 s and a final extension at 72°C for 5 minutes. The 10 *μ*L of PCR products was supplied with 1 *μ*L EvaGreen (Bio-Rad, USA) and then subjected to HRM in CFX96 real-time system C1000 Thermal Cycler (Bio-Rad, USA). The samples were first denatured at 95°C for 30 s and rapidly cooled to 40°C for 30 s and then melted from 55°C to 90°C with a 0.3°C/s ramp rate. Melting curves were analyzed with Bio-Rad Precision Melt Analysis software (Bio-Rad, USA). The sequences of the primers for rs6887695 were as follows: forward 5′- GGT AAG TCA GTT TGA GAG AAG CA -3′, reverse 5′- CTA GGT CAC AAG CGT AGT AAA TG -3′, and unlabeled C3-blocked probe 5′- TGT AGT GTA GTG GTC AAT AGT CTG GAT TT -3′.

### 2.3. Statistical Analysis

The SNP was analyzed for an association with the disease by means of comparison of the minor allele frequency (MAF) in patients and controls as well as the constancy of Hardy-Weinberg equilibrium using chi square test or Fisher's exact test. The magnitude of association was expressed as odds ratio (OR) with a 95% confidence interval (95% CI). *P* values less than 0.05 were considered statistically significant. 

## 3. Results

### 3.1. High-resolution Melting Analysis (HRMA) with Unlabeled Probe

**Figure 1:**
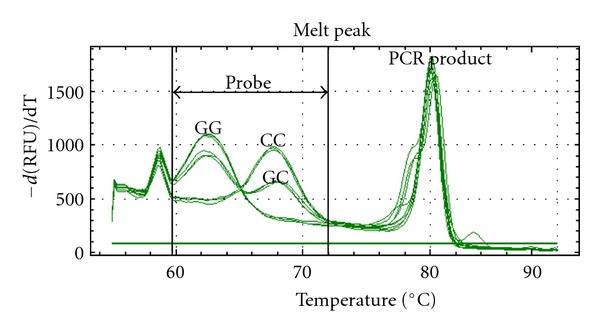
Derivative melting curves of unlabeled probes and amplicon for genotyping of SNP rs6887695. Three genotypes (GG, GC and CC) were discriminated as indicated in probe region.

**Figure 2:**
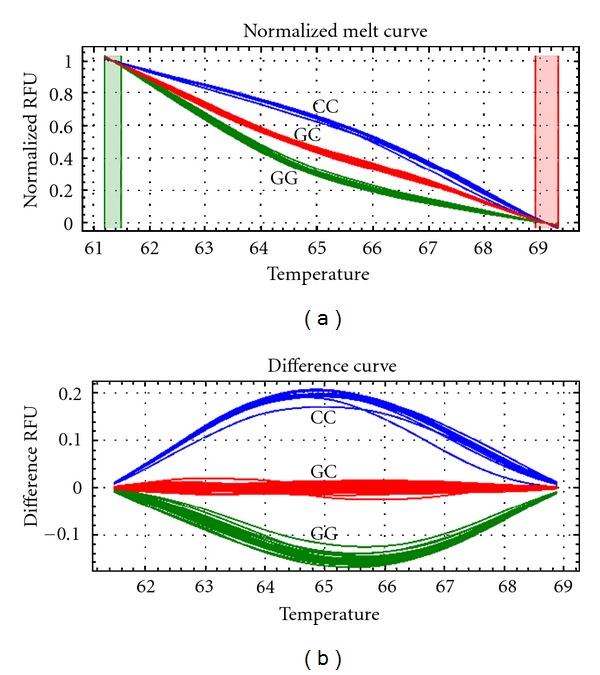
Normalized melting curves and difference curves of unlabeled probe region for genotyping of SNP rs6887695. (a) The melting curve was presented after normalization. Wild type GG genotype showed the lowest melting temperature since there was completely mismatch between the unlabeled probe and wild type. (b) The difference curves were obtained by subtracting each curve from one heterozygote (GC) curve. Three genotypes (GG, GC, and CC) were shown as indicated.

High-resolution melting analysis with unlabeled C3-blocked probe was used for genotyping. As shown in Figure 1, three genotypes (GG, GC, and CC) of SNP rs6887695 (G > C) were accurately distinguished by the derivative melting curves in the probe region. The derivative melting curves directly from the PCR products were not able to distinguish these genotypes (Figure 1, right part), and the sensitivity and accuracy of HRMA were dramatically improved using unlabeled probes (Figure 1, left part). After further analyzing on the normalized melting unlabeled probe region, we were able to discriminate all the genotypes clearly (Figure 2(a)). The difference curves generated by subtracting of one heterozygote curve displayed a much more clear view for genotype discrimination (Figure 2(b)). Then, this method was employed to screen our samples in the present study. 

### 3.2. Association of SNP rs6887695 with SLE

Table 1 shows IL-12b rs6887695 genotype and allele frequencies in SLE patients and healthy controls. Genotype frequencies were in Hardy-Weinberg equilibrium in the patients and controls. Statistically significant differences were observed in both genotype and allele frequencies for rs6887695 in the SLE patients as compared with the controls. Minor alleles of rs6887695 (*P* = 0.031, OR 0.78, [95% CI 0.63–0.98]) were found to be protective against SLE. 

### 3.3. Association of SNP rs6887695 with the Diagnostic Criteria of SLE

In order to further analyze the possible significance of IL-12b SNPs in SLE, we compared the genotype and allele frequencies of rs6887695 in different diagnostic criteria of SLE. Among the 11 criteria, serositis and neurological disorders in our study were in low incidence and thus were not subjected to statistical analysis.

Table 2 showed the analysis of IL-12b SNPs with the clinical symptoms of SLE. We found that rs6887695 was associated with the incidence of arthritis (*P* = 0.033), and minor alleles (C) was protective against arthritis (*P* = 0.013, OR = 0.65, 95% CI = 0.47–0.92). No obvious association was observed between IL-12b SNPs and the incidence of other clinical symptoms.

Table 3 showed the association of IL-12b SNPs with the laboratory parameters of SLE. Interestingly, IL-12b SNPs were associated with antinuclear antibody (*P* = 0.032). Minor alleles (C) of rs6887695 were found to exert protective effect on the abnormalities of antinuclear antibody (*P* = 0.022, OR = 0.68, 95% CI = 0.49–0.95). IL-12b SNPs were irrelevant to other laboratory parameters of SLE. 

## 4. Discussion

Genotyping is an elementary method for SNP analysis. High-resolution melting analysis (HRMA) is a cost-effective and high throughput method for SNP genotyping [22–24]. However, it is sometimes difficult to distinguish SNPs of A/T or G/C (G/C in our case), which make up about 16% of human SNPs, because such SNPs cause only almost undetectable Tm shift (less than 0.4°C). Unlabeled probe melting analysis is a modified HRMA in which a ~30 bp C3-blocked probe is used to target the SNP of interest during the melting. A single-base-pair difference in such a short probe could result in a significant Tm shift (e.g., 3-4°C). As shown in our study, we can discriminate almost all the SNPs in a more accurate and reliable manner using unlabeled probe melting analysis. Thus, the technology of HRMA with unlabeled probe is strongly recommended for studying genetic components of complex diseases such as SLE.

The association of IL-12b with several autoimmune diseases has been well revealed [5–9]. SLE is a typical chronic autoimmune disease caused by dysregulation of immune system. As a common subunit of IL-12 and IL-23, IL-12b could participate in regulation of several important autoimmune responses especially in Th1 cell maturation and Th17 cell development [1, 4]. However, there is little evidence to suggest that IL-12b was associated with disease risk of SLE up to now. In present study, we confirmed that SNP rs6887695 in the gene IL-12b was associated with SLE in the Mainland Chinese Han population, which replicated the findings from a GWAS study in Caucasian population [19], while in a previous study, two SNPs located in the promoter and 3′UTR region of IL-12b gene, respectively, exhibited no association with the susceptibility of SLE in Spanish population [25]. The discrepancy in these findings may be explained by the fact that different SNPs located on IL-12b gene were examined in these studies. The SNP rs6887695 was reported to be associated with psoriasis which is also caused by dysregulation of autoimmune system [9]. Exploring the association of IL-12b with disease risk of SLE in our and others' work could provide us with a common genetic background for autoimmune disease.

In this study, we also analyzed the association of SNP rs6887695 with different diagnostic criteria of SLE. The potential role of rs6887695 in SLE was further proved by its association with some of the clinical symptoms (arthritis) and laboratory parameters (antinuclear antibody) of SLE. The minor allele (C) of rs6887695 was found to be protective against such diagnostic criteria, indicating the aspects through which rs6887695 exerts protective effect on the risk of SLE. This also implies that, at least in Chinese mainland population, rs6887695 in IL-12b gene may not only be a risk factor specific to SLE, but also might be a contributor to the severity of this autoimmune disease. However, it is not clear why rs6887695 has association with such particular parameters. In the future, the underlying mechanism of rs6887695 in SLE or other autoimmune diseases remains to be more extensively studied.

## Figures and Tables

**Table 1:** Genotype and allele frequencies of IL-12b SNP rs6887695 in SLE cases and controls*.

		Genotype frequency, no. (%)				Allele frequency, no. (%)			
SNP, population	No. of subjects	Major homozygote	Hetero-zygote	Minor homozygote	*P*	Major allele	Minor allele	*P*	OR (95% CI)
Genotype or allele		GG	GC	CC		G	C		
Cases	297	107 (36.0)	153 (51.5)	37 (12.5)	0.019	367 (61.8)	227 (38.2)	0.031	0.78 (0.63–0.98)
Controls	351	114 (32.5)	164 (46.7)	73 (20.8)		392 (55.8)	310 (44.2)		

*SNP: single-nucleotide polymorphism; SLE: systemic lupus erythematosus; OR: odds ratios; 95% CI: 95% confidence interval.

**Table 2:** Association of IL-12b SNP rs6887695 with clinical symptoms in SLE patients*.

		Genotype frequency, no. (%)				Allele frequency, no. (%)			
SNP, population	No. of subjects	Major homozygote	Hetero-zygote	Minor homozygote	*P*	Major allele	Minor allele	*P*	OR (95% CI)
Genotype or allele		GG	GC	CC		G	C		
Total cases	297	107	153	37		367	227		
Rash									
Positive	161	59	83	19	0.923	201	121	0.728	0.94 (0.68–1.31)
Negative	136	48	70	18		166	106		
Photosensitivity									
Positive	90	32	47	11	0.987	111	69	0.969	1.01 (0.70–1.44)
Negative	207	75	106	26		256	158		
Arthritis									
Positive	181	74	90	17	0.033	238	124	0.013	0.65 (0.47–0.92)
Negative	116	33	63	20		129	103		
Oral ulcer									
Positive	74	27	39	8	0.884	93	55	0.761	0.94 (0.64–1.38)
Negative	223	80	114	29		274	172		

*SNP: single-nucleotide polymorphism; SLE: systemic lupus erythematosus; OR: odds ratios; 95% CI: 95% confidence interval.

**Table 3:** Association of IL-12b SNP rs6887695 with laboratory parameters in SLE patients*.

		Genotype frequency, no. (%)				Allele frequency, no. (%)			
SNP, population	No. of subjects	Major homozygote	Hetero-zygote	Minor homozygote	*P*	Major allele	Minor allele	*P*	OR (95% CI)
Genotype or allele		GG	GC	CC		G	C		
Total cases	297	107	153	37		367	227		
Hematological disorders									
Positive	187	63	98	26	0.429	224	150	0.216	1.24 (0.88–1.76)
Negative	110	44	55	11		143	77		
Proteinuria									
≥0.5 g/24 h	101	35	52	14	0.851	122	80	0.617	1.09 (0.77–1.55)
<0.5 g/24 h	196	72	101	23		245	147		
Antinuclear antibody									
≥1 : 320	168	67	87	14	0.032	221	115	0.022	0.68 (0.49–0.95)
<1 : 320	129	40	66	23		146	112		
Anti-DNA									
Positive	189	72	97	20	0.352	241	137	0.191	0.80 (0.57–1.12)
Negative	108	35	56	17		126	90		
Anti-nRNP									
Positive	81	31	41	9	0.846	103	59	0.581	0.90 (0.62–1.31)
Negative	216	76	112	28		264	168		
Anti-Smith									
Positive	68	24	37	7	0.783	85	51	0.845	0.96 (0.65–1.43)
Negative	229	83	116	30		282	176		
Anti-Ro (SSA)									
Positive	118	43	61	14	0.968	147	89	0.837	0.97 (0.69–1.35)
Negative	179	64	92	23		220	138		
Anti-La (SSB)									
Positive	72	25	38	9	0.963	88	56	0.848	1.04 (0.71–1.53)
Negative	225	82	115	28		279	171		
C3 and C4									
Decrease	133	49	79	15	0.399	177	109	0.960	0.99 (0.71–1.38)
Normal	164	58	74	22		190	118		

*SNP: single-nucleotide polymorphism; SLE: systemic lupus erythematosus; OR: odds ratios; 95% CI: 95% confidence interval.
